# The Role of Major Inflammatory Biomarkers in the Pathogenesis of Atrial Fibrillation

**DOI:** 10.19102/icrm.2022.13125

**Published:** 2022-12-15

**Authors:** Saira Rafaqat, Shaheed Afzal, Huma Khurshid, Sanober Safdar, Sana Rafaqat, Simon Rafaqat

**Affiliations:** ^1^Lahore College for Women University, Lahore, Pakistan; ^2^Punjab Institute of Cardiology, Lahore, Pakistan; ^3^Imperial College of Business Studies, Lahore, Pakistan; ^4^Forman Christian College (A Chartered University), Lahore, Pakistan

**Keywords:** Atrial fibrillation, CD40 ligand, inflammatory biomarkers, matrix metalloproteinase-9, monocyte chemoattractant protein-1

## Abstract

Many studies have reported a relationship between inflammation and atrial fibrillation (AF). According to the literature, inflammation is the key component in pathophysiological processes during the development of AF; the amplification of inflammatory pathways triggers AF, and, at the same time, AF increases the inflammatory state. The plasma levels of several inflammatory biomarkers are elevated in patients with AF; therefore, inflammation might contribute to both the maintenance and occurrence of AF and its thromboembolic complications. Numerous inflammatory markers have been linked to AF, including CD40 ligand, fibrinogen, matrix metalloproteinase (MMP)-9, monocyte chemoattractant protein-1, myeloperoxidase, plasminogen activator inhibitor-1, and serum amyloid A. There are many pathophysiological aspects of AF that are linked to these inflammatory biomarkers, including atrial structural remodeling and atrial dilatation, increased atrial myocyte expression, fluctuations in calcium cycling, cardiac remodeling promotion, increased cardiac myocyte proliferation and terminal differentiation, production of several MMPs, the pathogenesis of atherosclerosis and cardiomyocyte apoptosis, an increased degree of fibrosis in atrial myocardium, and the progression and development of atherogenesis and atherothrombosis. The present review article aims to provide an updated overview and focus on the basic role of different biomarkers of inflammation in the pathophysiological aspects of the pathogenesis of AF.

## Introduction

Atrial fibrillation (AF) is the most common abnormal heart rhythm disorder. Globally, >33.5 million people are affected by AF.^1,2^ The clinical consequences of AF include a reduced quality of life as well as increased mortality from cardiovascular disease (CVD).^3^ Inflammation is a complex dynamic protective response to cell injury, microbial infection, trauma, or toxins in the vascularized tissues. Moreover, biomarkers provide a powerful and dynamic tool to grasp the spectrum of inflammatory diseases with applications in observational and analytic epidemiology, clinical trials in various populations, and screening with diagnosis and prognosis.^4^

Guo et al. has previously covered the numerous inflammatory markers that have been linked to AF, including C-reactive protein (CRP), tumor necrosis factor (TNF), interleukin (IL)-2, IL-6, and IL-8. There are many proposed mechanisms that are related to the prothrombotic AF state with inflammation, such as increased platelet activation, endothelial activation/damage, production of tissue factor from monocytes, and increased expression of fibrinogen. Also, inflammatory markers have an impact on the clinical presentation as well as the outcome of patients with AF.^5^ Zhang et al. reported a direct relationship between AF and inflammation,^6^ and the predictors of AF recurrence have also been validated by myocardial fibrosis and inflammatory biomarkers.^7^

Likewise, Zacharia et al. also reported the role of inflammatory biomarkers in AF. Inflammation is a key component in pathophysiological processes during the development of AF; the amplification of inflammatory pathways triggers AF, and, at the same time, AF increases the inflammatory state. Certainly, the plasma levels of several inflammatory biomarkers are elevated in patients with AF.^8^ This review article only focuses on the role of major biomarkers of inflammation—such as cluster of differentiation 40 ligand (CD40L), endothelin-1, fibrinogen, matrix metalloproteinase (MMP)-9, monocyte chemoattractant protein (MCP)-1, myeloperoxidase (MPO), plasminogen activator inhibitor (PAI)-1, and serum amyloid A (SAA)—in the progression and development of AF.

## Methods

Many studies have reported the relationship between inflammation and AFs; however, this review article discusses only major inflammatory biomarkers that have not been previously reported on to explain their role in the pathogenesis of AF, provides an overview and focuses on the basic role of different biomarkers of inflammation, and highlights pathophysiological aspects in the pathogenesis of AF. There are many inflammatory biomarkers, but this review article focused only on CD40L, fibrinogen, MMP-9, MCP-1, MPO, PAI-1, and SAA.

Different databases, including Google Scholar, PubMed, and ScienceDirect, were used to review the literature. The date of the last literature search was March 20, 2022. Many keywords were used for searching the literature, including “atrial fibrillation,” “inflammatory biomarkers,” “CD40 ligand,” “fibrinogen,” “matrix metalloproteinase-9,” “monocyte chemoattractant protein-1,” “myeloperoxidase,” “plasminogen activator inhibitor-1,” and “serum amyloid A.” The language of clinical studies was restricted to English. We did not place limits on the publication time, although more recent studies were favored. References from relevant articles were reviewed, and related articles were identified.

### Role of major inflammatory biomarkers in atrial fibrillation

CD40L, fibrinogen, MMP-9, MCP-1, MPO, PAI-1, and SAA play pathophysiological roles in AF, as explained in **Table 1**.

#### CD40 ligand

CD40 is a costimulatory protein present on antigen-presenting cells that require it for their activation.^9^ CD40 is a transmembrane protein; together with its ligands, including CD40L (trimeric) or CD154, it is a member of the TNF receptor superfamily, and it forms a trimer, a high order of collection of the protein obligatory for optimal signaling.^10,11^ Immunohistochemical studies have explained that CD40, which is present on the surface of B lymphocytes, was originally identified and employed as an antibody capable of detecting a 50-kDa protein. In the scientific community, the CD40/CD40L receptor/ligand dyad has received growing attention.^12,13^

CD40L is also a pro-inflammatory marker released by platelet activation and involved in pro-inflammatory transcriptional pathway regulation, including inflammatory signaling, as well as myocardial oxidative stress.^14,15^ Additionally, inflammation processes play a significant role in the pathogenesis of CVDs. Various biomarkers of inflammation provide additional prognostic value in predicting cardiovascular events. The CD40/CD40L system plays a role in the pro-inflammatory and prothrombotic processes of CVDs and creates a link between these pathways. Numerous diseases, including heart failure, obesity, diabetes, and metabolic syndrome, involve elevated levels of soluble CD40L (sCD40L).^16^

Additionally, elevated CD40L levels are reported in AF patients, which suggests an explanation for the increase in thrombosis during this arrhythmia.^17^ Further, the study by Antoniades et al. demonstrated a higher risk of developing AF after off-pump coronary artery bypass grafting surgery, which is associated with preoperative elevated levels of sCD40L.^18^ In the same context, Bozçalı et al.’s study described the relationship between a prothrombotic, CD40L as a pro-inflammatory molecule, and lone AF for the first time. Moreover, these authors suggested that CD40L levels play an important role in the progression of lone AF. Regular clinical follow-up is required in the studied subjects because of the increased risk of CVDs, which is determined by increased CD40L levels.^19^

Blann et al.’s study determined that sCD40L levels were marginally elevated in AF patients relative to levels of P-selectin as well as von Willebrand factor.^20^ Similarly, Hammwöhner et al.’s study investigated atrial clot development in persistent AF patients, revealing the consequence of platelet CD40L expression and its relationship with inflammatory markers. An elevated expression of CD40L was observed in AF patients with atrial thrombi; even 5 weeks after effective cardioversion, the quantity of platelet CD40L expression remained increased. The authors concluded that this might increase the risk of atrial thrombus formation.^21^ Finally, Duygu et al.’s study demonstrated that elevated levels of sCD40L could predict thrombus formation as well as stroke in AF patients prospectively.^22^

Ferro et al.’s study reported elevated levels of sCD40L in non-valvular AF (NVAF) patients, which is a predictor of vascular events. Therefore, these authors suggested that clinical progression occurred due to enhanced platelet activation. The prediction of cardiovascular events was also enhanced due to the levels of plasma sCD40L (>4.76 ng/mL) in NVAF patients.^23^ Various studies have shown a confounding issue as to whether increased levels of sCD40L are due to AF and linked to cardiovascular comorbidities and have suggested an association between comorbidities and arrhythmia or a third and common driving factor. Evidence suggests that raised sCD40L levels and platelet activation are causes of AF onset, explaining their role in the arrhythmia itself.^24,25^ Furthermore, Cohoon et al.’s study revealed that patients with NVAF had higher sCD40L levels compared to normal sinus rhythm (SR) controls, but for only up to 1 year after the development of dysrhythmia. To assess the development or recurrence of asymptomatic NVAF, an sCD40L level of 552 pg/mL could be helpful.^26^

In contrast, Lip et al.’s study reported that CRP level is connected to the threat and risk factors of stroke as well as its prognosis, which includes vascular events and mortality. Maximal CRP levels have been seen in AF patients at modest-to-high risk for stroke. However, the authors did not report clear relationships with sCD40L, also suggesting an inadequate understanding of platelet activation in thrombogenesis in AF. In the risk stratification of factors influencing AF, CRP level could be used for further study.^27^

#### Fibrinogen

Fibrinogen is a major structural component of a clot synthesized by the liver and located on chromosome 4 with 3 different genes that encode for fibrinogen synthesis. Fibrinogen is a 340-kDa hexametric plasma glycoprotein whose plasma concentration is approximately 200–400 mg/dL and whose plasma half-life is 3–4 days; a minimum level of 100 mg/dL is required to maintain hemostasis.^28,29^

Moreover, an elevated level of fibrinogen indicates the presence of inflammation and is recognized as a biomarker of inflammation that identifies individuals with an elevated risk for CVD disorders.^30^ Studies have proved that an increased blood content of fibrinogen could be caused by numerous inflammatory disorders.^31,32^

In the same context, Lip et al.’s study reported that increased fibrin D-dimer and fibrinogen concentrations in plasma are associated with AF. Patients with paroxysmal AF have intermediate levels of these biomarkers, which also places them at intermediate risk for thromboembolism.^27^ Further, Weymann et al. explained the connection of endothelial function, coagulation activation, and fibrinolytic markers with the occurrence of AF and clinical adverse events. In conclusion, the authors found a highly significant association of fibrinolytic markers, endothelial markers, and levels of coagulation (fibrinogen with a weighted mean difference of 0.43 and *P* < .001) with AF in AF patients compared to SR patients.^33^

Another study reported a significant difference in the prothrombotic state (irregularity of blood coagulation) when patients with permanent as well as paroxysmal AF were compared to those with persistent AF or SR controls. After 3 months of SR maintenance, cardioversion of persistent AF did not meaningfully modify indices of hypercoagulability, despite the return of atrial systole.^34^ Similarly, Mukamal et al. demonstrated that a greater risk of AF relates to a higher concentration of fibrinogen and lower levels of albumin, even though these components are related to the risk of CVDs. The authors’ findings support the suggestion that the etiology of AF is related to inflammation.^35^ In the same way, Wu et al.’s study highlighted the significant link between increased circulating hemostatic factors and AF.^36^ Hu et al. found for the first time that the A allele of *FGB* 455G*/*A was a risk factor for cardioembolic stroke (CES) in AF patients, probably by way of an elevated level of plasma fibrinogen.^37^

#### Monocyte chemoattractant protein-1

Chemokines including MCP-1 are produced nearby and released by numerous types of cells, such as macrophages, fibroblasts, and endothelial cells.^38^ Additionally, chemotaxis is involved in the recruitment of circulating monocytes in tissue inflammation. Monocytes play a major role in the pathophysiology of numerous CVDs, including myocardial infarction (MI), heart failure, and atherosclerosis. The pathological implication of chemotaxis by monocytes has been implied not only in atherosclerotic plaque formation but also in cardiac remodeling after myocardial injury.^39^

MCP-1 is a pro-inflammatory chemotactic cytokine that plays a role in thrombosis as well as atherogenesis, is upregulated in AF patients, and is involved in regulating the production of several MMPs. A 14% increased risk of CES had been associated with a genetically predetermined increased MCP-1 concentration.^40^

Likewise, there are numerous subtypes of the MCP-1 family, including MCP-1–induced proteins such as MCPIP, which is found in numerous human tissues. In the heart, the regulation of inflammatory responses through greater degradation of cytokine messenger RNAs as well as the IL-1–induced nuclear factor kappa B signaling pathway and inhibition of lipopolysaccharides are the primary functions of MCP. In the process of atherosclerosis formation, cardiomyocyte apoptosis (programmed cell death) and MCPIP-1 take on the roles of a transcription factor and differentiation factor in adipogenesis. In AF patients, the pathogenesis of atrial remodeling progression occurs due to elevated migratory activity in circulating and local monocytes.^41^

In the literature, Li et al.’s study revealed higher plasma levels of MCP-1 in lone AF patients compared to control subjects.^42^ In contrast, Georgakis et al. reported that AF was not associated with the genetic predisposition of elevated levels of MCP-1.^40^ Further, Abe et al.’s study explained that the degree of fibrosis suggests a deposition of connective tissues in atrial myocardium, which is associated with MCP-1 levels in epicardial adipose tissue dissected from the left atrial appendage in AF subjects. It was suggested by Abe et al. that atrial remodeling is due to the tissue local chemokine expression in the left atrium and clinical AF development,^43^ while another study reported no relationship between the proportions of monocyte subsets and left atrial dimension.^44^

#### Matrix metalloproteinase-9

MMPs are a family of proteolytic enzymes that also adjust extracellular matrix turnover in concert with tissue inhibitors of metalloproteinases (TIMPs).^45,46^ In the pathogenesis of CVDs, extracellular matrix degradation by MMPs is linked to dilated cardiomyopathy, restenosis, MI, and atherosclerosis. Furthermore, MMP-9 has a role in myocardial matrix remodeling induced by ischemia–reperfusion, which could be a target for acute ischemic myocardial injury inhibition as well as for providing support in treatment.^47^

During AF, MMP-9 contributes to atrial dilatation and atrial structural remodeling.^47^ In the same way, Sonmez et al.’s study described the progression of AF, which is associated with novel fibrotic and inflammatory markers, such as MMP-9.^48^ Moreover, Huxley et al. revealed that the increased risk of AF is independently linked to elevated levels of MMP-9.^49^ Li et al.’s study also confirmed that the development of AF is related to the levels of MMP-9. Remarkably, in the development of idiopathic AF, the MMP-9 levels progressively elevate from paroxysmal AF through persistent AF to permanent AF.^50^

Similarly, Lewkowicz et al. concluded that the occurrence and maintenance of AF are due to a marker of atrial remodeling, which might be MMP-9, and could be a potential therapeutic target by the regulation of the extracellular collagen matrix.^51^ Further, Kalogeropoulos et al.’s and Mukherjee et al.’s studies reported that the MMP superfamily includes various members, such as MMP-2, -3, and -7, while TIMPs such as TIMP-1, -2, and -3 have a strong link with the occurrence of AF.^52,53^

Elsewhere, Anné et al.’s study reported that structural atrial remodeling is due to mitral valve disease in AF patients. AF did not contribute to changed fibrosis or MMP expression in the left atrial appendage. The link between AF and right atrial appendage fluctuations could be caused by a greater hemodynamic load due to tricuspid regurgitation in these patients.^54^ Likewise, Wu et al.’s study revealed the independent prognosticator of recurrent arrhythmia after catheter ablation in patients with persistent AF with serum MMP-9 level measurements.^55^

#### Myeloperoxidase

MPO is an enzyme released into the extracellular fluid during the inflammatory process and stored in the azurophilic granules of polymorphonuclear neutrophils and macrophages. It is involved in inflammation as well as oxidative stress and is a possible marker of plaque instability. In coronary heart disease patients, MPO is a valuable clinical tool in their assessment.^56^ Moreover, MPO is a product of systemic inflammation that plays a vital role in both the processes of oxidative stress and inflammation, which also promote the oxidation of lipoproteins.^57^

Rudolph et al.’s study not only highlighted the implications of neutrophil activation as a life-threatening pathophysiological condition of AF but also revealed that MPO, by oxidatively modifying protein residues, increases the fibrosis of atrial myocytes, which is causally associated with the initiation and perpetuation of AF.^58^ In the same way, Liu et al. explained the link between postoperative AF with MPO and the ability to predict postoperative AF, which was remarkably improved by adding pericardial MPO. Atrial electrical and structural remodeling constitute the physiological substrate for this arrhythmia in which MPO was related.^59^

Holzwirth et al.’s study also concluded that increased MPO in AF could originate from the left atrium. The missing association of MPO levels with AF progression or recurrence supports the theory that MPO release due to unknown stimuli induces fibrotic processes and is not a surrogate marker of fibrotic remodeling. The reduction of peripheral MPO levels through renin–angiotensin system antagonists suggests a novel mechanism of their protective effects regarding AF incidence. The identification and inhibition of momentarily unknown stimuli promoting left atrial MPO release is a promising therapeutical target.^60^

Rodionova et al.’s study suggested that increased levels of MPO (odds ratio, 1.115; 95% confidence interval, 1.007–1.841; *P* < .001) are associated with a greater risk of AF. MPO could be a possible significant factor for therapy optimization in high-risk ST-segment–elevation MI patients.^61^ In the same context, Li et al.’s study concluded that elevated MPO levels are associated with an increased risk of AF recurrence after catheter ablation.^62^ Similarly, Pauklin et al. stated that MPO might be a clinically valid prognostic marker for the assessment of AF recurrence after rhythm control therapy.^63^

#### Plasminogen activator inhibitor-1

PAI-1 plays an important role in modifying fibrinolysis and extracellular matrix remodeling and is also the primary inhibitor of 4 serine peptidase inhibitors. It binds and deactivates tissue plasminogens, such as tissue-type plasminogen activator (tPA) and urokinase plasminogen activator (uPA), which play a role in fibrin-regulating activity and intravascular plasminogen activation. Among these, the uPA receptor can alter its actions on different cells. PAI-1 can prevent intravascular fibrinolysis as well as cell-associated proteolysis.^64^

PAI-1 has a role in CVD outcomes as well as in increasing the mortality rate.^65^ In addition, various cross-sectional studies have demonstrated that tPA and PAI-1 concentrations are elevated in AF patients.^66,67^ However, Mulder et al.’s study reported that PAI-1 and tPA levels were not linked to the onset of AF.^68^ On the other hand, another study revealed that plasminogen activator levels after artery bypass surgery may be considered an independent predictor of AF. These authors further suggested that finding drugs able to decrease the levels of PAI-1 will ultimately reduce the burden of AF.^69^

Similarly, systemic inflammation as well as endothelial damage or dysfunction are early consequences of AF and could be attributed to insulin resistance, inflammation, fibrinolysis, endothelial damage, and/or hypertension.^70^ As a result, inflammation plays a major part in the progression of AF and might be responsible for the rise of many associated conditions, such as coronary artery diseases, hypertension, and obesity.^71^ Additionally, these diseases through fibrosis could be associated with structural remodeling of the atria, which might even encourage electrical remodeling of the atria and eventually result in AF.^68^

#### Serum amyloid A

The acute-phase protein (APP) SAA is a clinically useful inflammatory marker also linked to an increased risk of cardiovascular events.^71^ Moreover, chronically increased SAA levels could contribute to physiological processes, which may lead to characteristics of atherosclerosis, including endothelial dysfunction, and an early predictive event in the development of CVDs.^72–75^ SAA is a sensitive marker of inflammation, and higher levels of SAA were recorded in study subjects with acute and chronic inflammation. Long-term and repeated infections cause a persistent elevation in SAA. In addition, Cheng et al. confirmed elevated levels of SAA as a type of inflammatory ctyokine finding in AF patients for the first time. However, the reason for the increased SAA concentration in AF remains unidentified. Further, the inflammatory state could reflect elevated SAA levels, which could promote the occurrence and persistence of AF. In addition, in patients with permanent or persistent AF, SAA levels were significantly higher than those in patients with paroxysmal AF.^76^

In the same way, Barassi et al.’s study suggested conserved left ventricular ejection fraction, SAA level, and CRP level to be independent predictors of the AF subacute recurrence rate, whereas N-terminal pro-brain natriuretic peptide (NT-proBNP) was not linked to arrhythmic outcome, which also reflects the hemodynamic changes secondary to arrhythmia presence. In predicting the AF recurrence rate, the simultaneous determination of SAA and high-sensitivity CRP (hs-CRP) levels has a very high sensitivity (100%).^77^

Furthermore, Negreva et al.’s study determined that the clinical manifestation of paroxysmal AF is linked to an inflammatory response characterized by specific dynamics in the plasma concentrations of APPs, hs-CRP, and SAA. Their elevated values were measured still in the early hours of the rhythm disorder (around the eighth hour) and persisted after SR restoration before subsiding slowly over time. The characteristic features of these changes suggest that the APP response is closely related to the initiating mechanisms of paroxysmal AF.^78^

## Discussion

**Figure 1:**
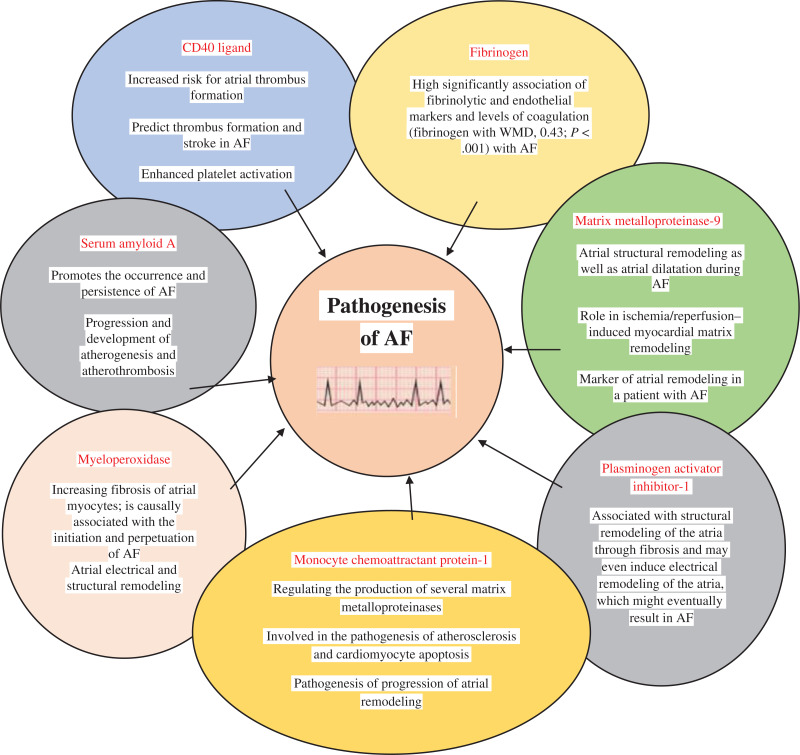
Relevance of each biomarker of inflammation in atrial fibrillation. *Abbreviations:* AF, atrial fibrillation; AF, atrial fibrillation; WMD, weighted mean difference.

This review article discusses the role of certain major biomarkers of inflammation (CD40L, fibrinogen, MMP-9, MCP-1, MPO, PAI-1, SAA) in the pathogenesis of AF, as explained in **Figure 1**. Many pathophysiological aspects of AF arise due to these inflammatory biomarkers, including atrial structural remodeling and atrial dilatation, increased atrial myocyte expression, fluctuations in calcium cycling, cardiac remodeling promotion, increases in cardiac myocyte proliferation and terminal differentiation, the production of several MMPs, the pathogenesis of atherosclerosis and cardiomyocyte apoptosis, an increased degree of fibrosis in atrial myocardium, and the progression and development of atherogenesis and atherothrombosis.

In the same way, Zhou and Dudley’s study found various mechanisms of inflammation-related AF involved the initiation of early and late afterdepolarization, enhanced fibrosis, inflammation-induced alternations of electrophysiological properties, and cardiac structure remodeling. These inflammatory factors induce the occurrence of ectopic activity and re-entry, which contribute to the initiation and maintenance of AF.^79^ Furthermore, Harada et al. also observed that inflammation may be associated with various pathological processes, including apoptosis, fibrosis, and oxidation stress, that promote AF substrate formation. Endothelial dysfunction, coagulation cascade activation, and platelet activation were related to inflammation, which led to thrombogenesis. Therefore, inflammation might contribute to both the maintenance and occurrence of AF and its thromboembolic complications.^80^

Pericarditis and myocarditis are local inflammatory conditions linked to the increased incidence of AF.^81^ Consistent with local inflammation contributing to arrhythmia, immune cells infiltrate the atria of AF patients,^82^ and activation of leukocytes is elevated in patients with perioperative AF.^83^ As an example, research found that CD45^+^ lymphocyte counts are higher in AF patients and CD68^+^ macrophage counts are higher in the atria of AF patients compared to in the control group.^84^ This suggests a link between inflammation and AF by immune cell infiltration into the atria.

There are various mechanisms for the onset and maintenance of AF, which involve electrical and structural remodeling to perpetuate AF. Atrial fibrosis is caused by structural remodeling and closely related to inflammation. Moreover, in the atria of AF subjects, inflammatory infiltration had been identified as affecting signaling pathways for AF development.^80,85^

Similarly, numerous studies have reported that higher levels of inflammatory markers, such as IL-6, TNF, CRP, heat shock protein β1, IL-8, and the neutrophil-to-lymphocyte ratio (NLR), are present in AF patients compared to SR controls. According to the literature, there are many biomarkers of inflammation.^79,86^ Hijazi et al. also concluded that, in AF patients on anticoagulation, after accounting for clinical risk factors and other biomarkers, biomarkers of inflammation were significantly associated with an increased risk of mortality. However, no associations with the risk of stroke or major bleeding were found.^87^

Korantzopoulos et al. also presented in detail a comprehensive review of AF and inflammation. The pathophysiology of atrial remodeling is implicated by inflammation, which is confirmed by experimental and clinical data. There is a complex mechanistic relationship between inflammation and atrial remodeling, and diverse underlying diseases and conditions could affect these pathways. In AF development, disease perpetuation, recurrence, thromboembolic complications, and total AF burden are associated with inflammatory markers. In addition, various agents with direct or indirect/pleiotropic anti-inflammatory properties are being tested in AF clinical as well as experimental settings.^88^

Jabati et al. conducted a baseline analysis of circulating biomarkers in a group of patients with AF prior to ablation and found that different prothrombotic, inflammatory, and collagen turnover biomarkers (PAI-1, CD40L, nucleosomes, CRP, procollagen III N-terminal propeptide, and procollagen III C-terminal propeptide) are elevated in AF. It would be interesting to correlate the levels of these biomarkers with clinical outcomes and treatment in this population.^89^

Also, Zhang et al.’s study provides further clinical epidemiological evidence that systemic inflammatory status correlates with AF. The systemic inflammation score, as an index to evaluate the intensity of systemic inflammatory status, could be useful for the early prediction of AF development and understanding of AF.^6^ In the same context, Luo et al.’s study concluded that the raised systemic immune–inflammation index again was linked to an increased risk of the postoperative recurrence of AF and independently predicted the late recurrence of AF following the CryoMAZE procedure concomitant with mitral valve surgery.^90^

Likewise, Karavelioğlu et al.’s study reported that increased NLR is a marker of increased inflammation and may serve as a simple, cheap, and readily available predictor of recurrence during the long-term follow-up of patients admitted with acute AF who were successfully converted to SR with amiodarone.^91^ Similarly, NLR, an emerging marker of inflammation, was independently associated with the presence of left atrial thrombus in patients with non-valvular AF.^92^ Correspondingly, Marcus et al. concluded that AF was independently linked to inflammation. Differences in transcardiac gradients suggest that AF results in the sequestration of inflammatory cytokines in the heart.^93^ Additionally, an elevated plasma CRP concentration per se does not increase the AF risk, and values obtained for CCL2 suggest that inflammation is probably a consequence of AF. The authors also suggest that the effect of the duration of the AF episode should be further studied in the assessment of the actual role of inflammation.^94^

Ishii et al. also assessed whether the degree of atrial inflammation was associated with a proportional increase in the inhomogeneity of atrial conduction and AF duration, which may be a factor in the pathogenesis of early postoperative AF. Their study suggests that anti-inflammatory therapy has the potential to decrease the incidence of AF after cardiac surgery.^95^

In the same way, Pauklin et al.’s study revealed elevated levels of MPO, NT-proBNP, hs-CRP, and galectin-3 in AF subjects compared to healthy controls. Both MPO and hs-CRP concentrations were independently associated with AF, and lower baseline MPO levels were associated with better long-term outcomes after AF ablation or electrical cardioversion. These results indicate that inflammatory and fibrotic processes play an important role in the development of AF. MPO might be a clinically valid prognostic marker for the assessment of AF recurrence after rhythm control therapy and necessitates further studies to confirm this hypothesis.^63^

AF shows a hypofibrinolytic state caused by elevated PAI-1 levels with no increase in the concentration of plasmin–antiplasmin complexes. Elevated plasma D-dimer levels suggest the presence of increased intravascular thrombogenesis. This may contribute to an increased risk of thrombosis.^96^

Furthermore, Patel et al. presented a detailed understanding of inflammation, which has a significant role in the initiation and perpetuation of AF and the prothrombotic state linked to AF. Inflammatory biomarkers (CRP and IL-6) are associated with the future development, recurrence, and burden of AF and the likelihood of successful cardioversion. Therapies directed at attenuating the inflammatory burden appear promising. Animal and clinical studies have evaluated statins, angiotensin-converting enzyme inhibitors/angiotensin-II receptor blockers, and corticosteroids for the treatment or prevention of AF.^97^ Further studies are required to reveal practical therapeutic approaches to control these biomarkers of inflammation, which could ultimately help to reduce the association of inflammation with AF.

## Conclusion

Biomarkers of inflammation play a significant role in the development and progression of AF. Also, major biomarkers of inflammation, such as CD40L, fibrinogen, MMP-9, MCP-1, MPO, PAI-1, and SAA, play pathophysiological roles in AF.

## Figures and Tables

**Table 1:** Summary of Different Major Biomarkers of Inflammation in the Pathogenesis of Atrial Fibrillation

Study	Publication Year	Number of Patients	Patients or Clinical Setting	Markers	Main Findings
Choudhury et al.^17^	2007	59	—	CD40L	Plasma levels of sCD40L are raised in patients with AF and are related to levels of vascular endothelial growth factor, Ang-2, and tissue factor
Antoniades et al.^18^	2009	144	John Radcliffe Hospital, Oxford, England	CD40L	The greater risk of developing AF after off-pump coronary artery bypass grafting surgery is associated with preoperatively elevated levels of sCD40L
Bozçalı et al.^19^	2016	35	Cardiology Outpatient Clinic, Istanbul	CD40L	CD40L levels play an important role in the progression of lone AF
Blann et al.^20^	2008	—	—	sCD40L	sCD40L was marginally elevated in AF patients compared to P-selectin as well as von Willebrand factor
Hammwöhner et al.^21^	2007	—	—	CD40L	In AF patients, an elevated expression of CD40L was found in patients with atrial thrombi
Duygu et al.^22^	2008	44	Outpatients	sCD40L	Elevated levels of sCD40L can predict thrombus formation as well as stroke in AF prospectively
Ferro et al.^23^	2007	231	IV Division of Clinical Medicine	sCD40L	Enhanced levels of plasma sCD40L (>4.76 ng/mL) in patient with non-valvular AF
Cohoon et al.^26^	2016	109	Primary Care Internal Medicine Clinic during their annual medical examination	sCD40L	Non-valvular AF subjects had higher sCD40L levels compared to those of normal SR controls
Lip et al.^27^	1996	33	Hospital	Fibrin D-dimer and fibrinogen	Increased fibrin D-dimer and fibrinogen in plasma is associated with AF
Weymann et al.^33^	2017	—	—	Fibrinolytic, endothelial markers, and levels of coagulation	A highly significant association of fibrinolytic markers, endothelial markers, and levels of coagulation was present in patients with AF than in SR patients
Mukamal et al.^35^	2006	286	Validated nationwide registry of all hospitalizations	Fibrinogen and albumin	A greater risk of AF was related to a higher concentration of fibrinogen and lower levels of albumin
Hu et al.^37^	2017	479	Hospital of Zhejiang University	Plasma fibrinogen	The authors provide evidence for a potential association of *FGB* 455G/A polymorphism with an increased risk of CES in AF patients with low CHA_2_DS_2_-VASc scores
Li et al.^42^	2010	305	—	MCP-1	Higher plasma levels of MCP-1 in lone AF patients compared to control subjects
Georgakis et al.^40^	2019	67,162	—	MCP-1	AF was not associated with the genetic predisposition of elevated levels of MCP-1
Abe et al.^43^	2018	59	—	MCP-1	The degree of fibrosis deposition in the atrial myocardium was associated with MCP-1 levels in epicardial adipose tissue dissected from the left atrial appendage in AF subjects
Nakano et al.^47^	2004	25	Hospital	MMP-9	Expression of MMP-9 was increased in fibrillating atrial tissue, which may have contributed to atrial structural remodeling and atrial dilatation during AF
Sonmez et al.^48^	2014	52	Outpatient clinic	Serum levels of galectin-3, MMP-9, lipocalin-2 (Lcn2/NGAL), N-terminal propeptide of type III procollagen, hs-CRP, and neutrophil-to-lymphocyte ratio	Novel fibrosis and inflammation markers in AF are correlated with atrial remodeling
Huxley et al.^49^	2013	13,718	—	MMP-9	An increased risk of AF was independently linked to elevated levels of MMP-9
Li et al.^50^	2014	25 patients in each group	Outpatients or inpatients receiving care at Renmin Hospital of Wuhan University	MMP-9	In the development of idiopathic AF, the MMP-9 levels progressively elevated from paroxysmal AF through persistent AF to permanent AF
Kalogeropoulos et al.^52^	2011	175	Hospital-based	Serum levels of MMP-2, MMP-3, MMP-9, and TIMP-1	Serum TIMP-1 and MMP levels in specific subgroups of hypertensive patients with AF and SR differed; lower serum levels of TIMP-1 were related to increased AF incidence, whereas higher serum levels of MMP-3 and MMP-9 and lower levels of TIMP-1 were strongly associated with permanent AF
Mukherjee et al.^53^	2013	82	Hospital-based	MMP-9, MMP-3, and TIMP-4	MMP-9, MMP-3, and TIMP-4 independently predicted AF recurrence
Anné et al.^54^	2005	19	University Hospital Gasthuisberg, Leuven, Belgium	MMPs and their inhibitors (TIMPs)	AF did not contribute to changed fibrosis or MMP expression in the left atrial appendage
Wu et al.^55^	2016	58	Hospital-based	MMP-9	The serum MMP-9 level is an independent predictor of recurrent arrhythmia after catheter ablation in patients with persistent AF
Rudolph et al.^58^	2010	—	—	MPO	MPO, by oxidatively modifying protein residues and increasing fibrosis of atrial myocytes, is causally associated with the initiation and perpetuation of AF
Liu et al.^59^	2021	137	—	MPO	The link between postoperative AF with MPO and the ability to predict postoperative AF was remarkably improved by adding pericardial MPO values
Holzwirth et al.^60^	2019	181	Heart Center Leipzig	MPO	The pro-fibrotic enzyme MPO is generally higher in AF subjects irrespective of AF type, the presence of low-voltage area, or midterm rhythm outcome. The authors suggested that MPO could directly originate from the left atrium. RAS-A decreases peripheral MPO concentrations in AF subjects.
Rodionova et al.^61^	2022	233	Hospital	MPO	Increased levels of MPO are associated with a higher risk of AF. MPO could be a possible significant factor for therapy optimization in high-risk STEMI patients
Li et al.^62^	2013	288	Hospital	MPO	Elevated MPO levels led to an increased risk of AF recurrence after catheter ablation
Pauklin et al.^63^	2022	75	Tartu University Hospital, North Estonia Medical Centre, Estonia,	MPO	MPO might be a clinically valid prognostic marker for the assessment of AF recurrence after rhythm control therapy
Mulder et al.^68^	2018	8,265	—	Plasminogen activator inhibitor-1 and tissue-type plasminogen activator levels	Plasminogen activator inhibitor-1 and tissue-type plasminogen activator levels were not linked to the onset of AF
Pretorius et al.^69^	2007	253	Hospital-based	PAI-1	After artery bypass surgery, plasminogen activator levels were considered an independent predictor of AF
Cheng et al.^76^	2012	122	Beijing 301 Hospital	SAA, hs-CRP, IL-6, TNF-α	Cytokines such as SAA, hs-CRP, IL-6, and TNF-α are associated with the initiation and progression of AF
Barassi et al.^77^	2012	57	Hospital	NT-proBNP, SAA, CRP levels	In predicting AF recurrence, simultaneous determination of SAA and hs-CRP levels has a very high sensitivity (100%)
Negreva et al.^78^	2016	51	Hospital admission	hs-CRP, SAA, and fibrinogen	Paroxysmal AF is linked to dynamics in hs-CRP and SAA plasma levels. The results suggest that inflammation is closely related to the arrhythmia initiation.

*Abbreviations:* AF, atrial fibrillation; Ang-2, angiopoietin 2; CD40L, CD40 ligand; CES, cardioembolic stroke; hs-CRP, high-sensitivity C-reactive protein; IL-6, interleukin-6; MCP-1, monocyte chemoattractant protein-1; MMP-9, matrix metalloproteinase-9; MPO, myeloperoxidase; NT-proBNP, N-terminal pro-brain natriuretic peptide; RAS-A, renin–angiotensin system antagonists; SAA, serum amyloid A; sCD40L, soluble CD40 ligand; SR, sinus rhythm; STEMI, ST-segment–elevation myocardial infarction; TIMP, tissue inhibitor of metalloproteinases; TNF-α, tumor necrosis factor-α.
